# Impact of premenstrual tension syndrome on academic performance among female university students from the United Arab Emirates: A cross‐sectional study

**DOI:** 10.1002/hsr2.70124

**Published:** 2024-10-09

**Authors:** Eman Ibrahim Mohamed Omara, Rasha Aziz Attia Salama, Talaat Matter Tadross, Sirwan Khalid Ahmed, Mona Gamal Mohamed, Syed Masudur Rahman Dewan, Md. Rabiul Islam

**Affiliations:** ^1^ College of Medicine Ras al Khaimah Medical and Health Science University Ras Al Khaimah United Arab Emirates; ^2^ College of Nursing University of Raparin, Rania, Sulaymaniyah Kurdistan Region Iraq; ^3^ Department of Nursing Ras al Khaimah Medical and Health Science University Ras Al Khaimah United Arab Emirates; ^4^ Department of Pharmacy, School of Life Sciences United International University (UIU) Dhaka Bangladesh; ^5^ School of Pharmacy BRAC University Dhaka Bangladesh

**Keywords:** academic performance, female students, premenstrual dysphoric disorder, premenstrual tension syndrome

## Abstract

**Background and Aim:**

Premenstrual tension syndrome can vary in type and severity among females, potentially affecting their academic performance. This study aims to examine the prevalence and severity of premenstrual tension syndrome symptoms in female university students and their impact on academics.

**Methods:**

A cross‐sectional descriptive study was conducted among female university students from Ras Al Khaimah, UAE over 6 months, from January 1, 2022 to June 30, 2022. The updated premenstrual tension syndrome self‐rating scale questionnaire was used to assess the prevalence and severity of symptoms. A total of 251 respondents were included in the study. The chi‐square test was used to determine the association between PMT and academic performance.

**Results:**

The results showed that 78.9% of the participants experienced premenstrual tension syndrome, with 16.3% of them reporting premenstrual dysphoric disorder. The majority of participants reported mild to moderate symptoms, with anxiety and depressed mood being the most common psychological symptoms. In terms of behavioral symptoms, physical symptoms and feeling overwhelmed were the most prevalent. Furthermore, 90% of participants reported a negative impact on their academic performance, with paying attention in class being the most affected. The study also found that PMT disorder had a significant impact on physical activities and extracurricular activities.

**Conclusions:**

Our study showed a high prevalence of premenstrual disorders among female students and their influence on physical activity and extracurricular participation. The study highlights the importance of implementing therapies for PMT syndrome and providing support to improve academic performance and overall quality of life.

## 1 BACKGROUND

Premenstrual tension (PMT) syndrome is a common disorder that affects women during their menstrual cycle. PMT includes premenstrual syndrome (PMS) and premenstrual dysphoric disorder (PMDD).^1^ PMS refers to recurring symptoms that occur during the luteal phase of the menstrual cycle and disappear during the follicular phase.^2^, ^3^ These symptoms can vary in severity and greatly impact a woman's daily life and well‐being. It's important to note that the intensity and specific symptoms can differ among individuals. On the other hand, PMDD is a more severe manifestation of PMS that affects 3%–8% of women in their reproductive years.^4^ PMDD is characterized by significant impairment in interpersonal relationships, social functioning, and work or academic performance.^5^

The exact cause of PMT is not fully understood, but it is believed to be a complex condition involving a combination of biological, psychological, and social factors. Hormonal imbalances, particularly changes in estrogen and progesterone levels throughout the menstrual cycle, play a significant role in PMT. These hormonal fluctuations can affect neurotransmitter activity, leading to mood changes and other symptoms. Additionally, certain genetic factors may influence an individual's susceptibility to experiencing PMT symptoms. Psychological factors, such as stress, emotional sensitivity, and coping mechanisms, may also contribute to the severity and perception of PMT symptoms. Females with a history of mood disorders or psychological conditions may be more susceptible to PMT symptoms.^6^ Social factors, such as cultural influences, societal expectations, and interpersonal relationships, can also influence the experience of PMT symptoms. Stressful life events and a lack of social support may worsen the severity of symptoms.

The Diagnostic and Statistical Manual of Mental Disorders, 5th edition (DSM‐5), provided criteria for diagnosing PMDD. According to these criteria, in most menstrual cycles, an individual must experience at least five symptoms that must appear in the week before menses, improve within a few days after menses begin, and be minimal or absent in the week post‐menses. These symptoms include at least one affective symptom (such as mood swings, irritability, depression, or anxiety) and additional symptoms (like decreased interest in activities, difficulty concentrating, fatigue, changes in appetite or sleep, feeling overwhelmed, and physical symptoms like breast tenderness or bloating.^7^ The symptoms must cause significant distress or interfere with daily activities and are not merely an exacerbation of another disorder.^8^ Managing PMT involves a combination of pharmacological and nonpharmacological interventions. The choice of treatment depends on symptom severity and individual circumstances. Nonpharmacological interventions, such as lifestyle modifications, regular exercise, stress reduction techniques, and dietary changes, are crucial.^9^ Additionally, psychological therapies, such as cognitive‐behavioral therapy, are important in helping individuals develop effective coping strategies and address negative thought patterns associated with PMT symptoms.

It is essential to understand the impact of PMT on medical science students due to the unique demands of their academic curriculum and the potential consequences for their future careers in healthcare. PMT symptoms, including emotional instability, irritability, difficulties with concentration, and fatigue, can greatly impair students' cognitive functions.^10^ These cognitive impairments can lead to decreased academic performance due to difficulties in focusing on lectures, studying for exams, and completing assignments. The additional burden of PMS can exacerbate stress and anxiety, further impacting their ability to perform academically. Students may find it challenging to attend classes regularly, participate actively in practical sessions, or engage in group projects, all of which are crucial components of their education.^2^

Moreover, the physical symptoms of PMS, such as headaches, muscle pain, and general discomfort, can lead to absenteeism, reducing the time available for learning and mastering essential skills.^11^ The psychological symptoms, including mood swings and depression, can also diminish motivation and self‐esteem, making it harder for students to keep up with their coursework and maintain high academic standards.^12^

There is a research gap in the existing literature regarding the impact of PMT on academic performance among female students, specifically in the United Arab Emirates (UAE). Investigating the influence of PMT on healthcare students can provide valuable insights to develop appropriate support systems and resources. These measures may include counseling services, flexible academic accommodations, awareness programs, and creating a supportive learning environment. It is also important to raise awareness among faculty and peers about PMT and its potential effects on female students, fostering understanding and empathy. To the best of our knowledge, this study is the first of its kind to be conducted in Ras Al Khaimah, UAE. The primary objective of this study is to assess the prevalence of PMS and PMDD among female students at Ras Al Khaimah Medical and Health Science University (RAKMHSU). Additionally, the study aims to evaluate the severity of associated physical, psychological, and behavioral symptoms and to examine the impact of PMT on the academic performance of female students.

## 2 METHODS

### 2.1 Study design and participants

A cross‐sectional descriptive study was conducted at RAKMHSU over 6 months, from January to June 2022. The study included all four constituent colleges: RAK College of Medical Sciences, College of Dental Sciences, College of Pharmacy, and RAK College of Nursing. Female students from RAKMHSU who have completed at least one term at the university have gained valuable academic experience. Female students from various colleges, academic years, and nationalities were recruited for the study. The exclusion criteria for female participants included amenorrhea, irregular menstrual cycles, polycystic ovarian disease, pregnancy, breastfeeding, ongoing hormonal therapy, or the use of psychiatric medications. A consecutive sampling technique^13^ was employed to recruit all female students who met the inclusion criteria and were available for participation in the study. The minimum sample size, estimated to be 256, was determined using a margin of error of 5%, a confidence level of 95%, a population size of 758, and a response distribution of 50%.^13^ A total of 260 female students responded to the study, of whom 251 were included in the analysis due to their provision of complete questionnaire responses.

### 2.2 Data collection

An anonymous self‐rated questionnaire was used to collect data on participant characteristics and evaluate the prevalence and severity of PMT symptoms. The first part of the questionnaire gathered demographic information about the participants, including age, age at menarche, marital status, nationality, and the presence of PMT symptoms. This section also explored how PMT symptoms affected the participants' academic performance, including attendance, participation in physical activities, timely completion of assignments, exam performance, involvement in extracurricular activities, and attentiveness in class. The second part of the questionnaire used the updated PMT syndrome rating scale, which aligns with the DSM‐5 criteria for PMDD. In this study, the self‐rated scale resembled the observed PMTS and assessed 11 symptom domains. These domains included depressed mood, anxiety/tension, emotional instability, irritability/hostility, decreased interest in usual activities, difficulty concentrating, a significant lack of energy, a subjective feeling of being overwhelmed or out of control, physical symptoms, eating habits, and sleeping habits. Respondents rated their symptoms on a scale of 0–4 for eight domains, indicating no symptoms, doubtful, mild, moderate, or severe. The last two domains were scored from 0 to 2, indicating no symptoms, mild, or marked changes. The maximum total score was 36.^14^ To improve the accuracy and interpretability of PMT symptom severity, the overall scores were categorized into distinct levels: normal (0–10), mild (11–18), moderate (19–27), or severe (≥28).^8^

To assess the validity and reliability of the questionnaire, a pilot study involving 10 female students who were not part of the main study sample was conducted. The scale's Cronbach's alpha coefficient was found to be 0.75, indicating internal consistency among the items and contributing to the questionnaire's reliability in measuring the severity of PMT symptoms.

### 2.3 Ethical approval

The local institutional Research and Ethics Committee approval was obtained before the initiation of the study (RAKMHSU‐REC‐162‐2022/23‐UG‐M). Written informed consent was obtained from participants with assured confidentiality.

### 2.4 Statistical analysis

The data collected was entered into Microsoft Excel to ensure completeness. Afterwards, it was coded and imported into the statistical package for the social sciences (SPSS) version 25.0 for analysis. The characteristics of the respondents and their PMTS were calculated in terms of numbers and percentages. The average and standard deviation of the continuous variables were then calculated. To examine the relationship between the presence of PMT (Yes/No) and various aspects of the respondents' academic performance, the chi‐square test was utilized. Factors including attendance, engagement in physical activities, timely submission of assignments, performance in exams, involvement in extracurricular activities, and attentiveness in class were analyzed. The test of significance used was two‐sided and *p*‐value < 0.05 was considered statistically significant.

## 3 RESULTS

### 3.1 Characteristics of the study participants

The study included a total of 260 respondents, but 9 of them had incomplete responses. Therefore, the final number of participants included in the study was 251. The participants had a mean age of 17.9 ± 8.9, with the youngest being 17 years old and the oldest being 29 years old. The majority of the respondents were single (97.6%) and 50.2% were non‐Arab. A little over half of the participants (59%) experienced menarche before the age of 12. The overall prevalence of PMTS among the participants was 78.9%, while the prevalence of PMDD was 16.3% (Table 1).

**Table 1:** Characteristics of the study participants.

Variables	Frequency (*n*)	Percentage (%)
Age in years		
17–20	139	55.3
21–22	75	29.8
23–24	30	12
≥25	7	2.8
Mean ± SD	20.3 ± 2.07	
Age of menarche in years		
<12	148	59
≥12	103	41
Marital status		
Single	245	97.6
Married	6	2.4
Nationality		
Local	50	19.9
Arab	75	29.9
Non‐Arab	126	50.2
PMT symptoms		
No	53	21.1
Mild	71	28.3
Moderate	86	34.3
Severe	41	16.3

Abbreviations: PMT, premenstrual tension; SD, standard deviation.

### 3.2 Changes in psychological, behavioral, and physical symptoms

**Figure 1:**
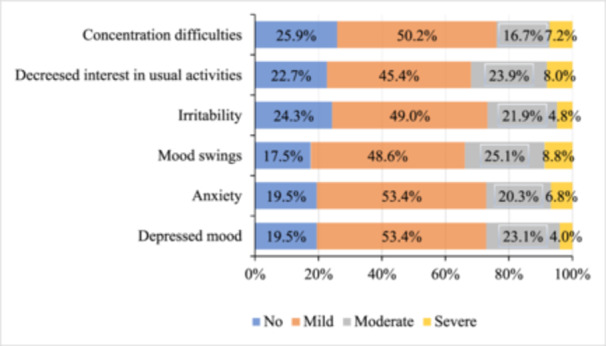
Changes of psychological symptoms due to premenstrual tension syndrome among the respondents.

Figure 1 reveals the distribution of psychological symptoms due to PMT syndrome among female students, highlighting that mild symptoms are the most prevalent. Specifically, over half of the students reported mild concentration difficulties (50.2%), with moderate and severe difficulties less common (16.7% and 7.2%, respectively). Similarly, nearly half experienced mild decreases in interest in usual activities (45.4%), with moderate and severe decreases reported by 23.9% and 8.0%. Mild irritability was noted by 49.0% of students, while moderate and severe irritability were reported by 21.9% and 4.8%, respectively. For mood swings, 48.6% of students experienced mild symptoms, with moderate and severe mood swings reported by 25.1% and 8.8%. Over half of the students experienced mild anxiety (53.4%), with moderate and severe anxiety reported by 20.3% and 6.8%. Additionally, a mild depressed mood was reported by 53.4% of students, with moderate and severe depressed moods noted by 23.1% and 4.0%. Overall, the data indicates that while mild symptoms of PMS are most common, moderate and severe symptoms are also present among the surveyed students.

**Figure 2:**
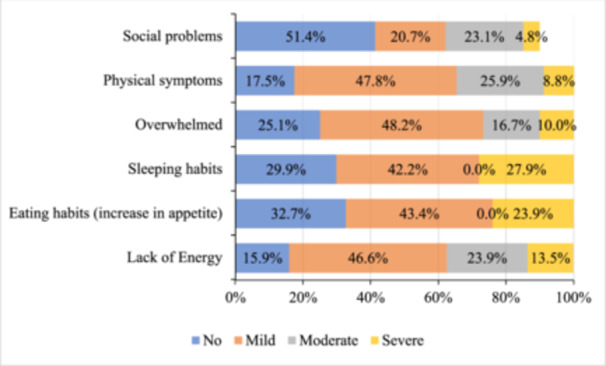
Changes of behavioral and physical symptoms due to premenstrual tension syndrome among the respondents.

Regarding behavioral and physical symptoms, Figure 2 shows that among respondents, those with mild symptoms most frequently reported a lack of energy (46.6%), with this issue being less common in those with moderate (23.9%) and severe symptoms (13.5%). An increase in appetite was reported by 43.4% of those with mild symptoms and 23.9% with severe symptoms, but none of the respondents with moderate symptoms experienced this change. Changes in sleeping habits were noted by 42.2% of individuals with mild symptoms and 27.9% with severe symptoms, while no changes were reported among those with moderate symptoms. Feeling overwhelmed was the most prevalent symptom in mild cases, affecting 48.2%, whereas social problems were the least common at 20.7%. Conversely, physical symptoms were most frequently reported by those with moderate symptoms (25.9%), and severe social problems were the least common (4.8%).

### 3.3 Impact on academic performance

**Figure 3:**
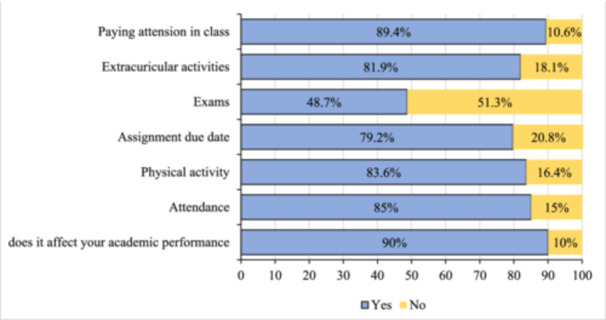
The impact of premenstrual tension syndrome on different aspects of the respondents' academic performance.

Figure 3 shows that 90% of the surveyed respondents reported that PMT affected their academic performance. The most significantly impacted areas were the ability to concentrate in class (89.4%), class attendance (85%), limitations in physical activities (83.6%), participation in extracurricular activities (81.9%), meeting assignment deadlines (79.2%), and performance in examinations (48.7%).

Table 2 illustrates the relationship between premenstrual tension syndrome (PMT) and various aspects of academic performance. The chi‐square test of significance shows that PMT significantly affects physical activities (*p* = 0.024), with 86.3% of those with PMT reporting difficulties compared to 69.4% without. It also significantly impacts extracurricular activities (*p* = 0.035), with 84.2% of those with PMT reporting difficulties compared to 69.4% without. The impact on exam performance approached significance (*p* = 0.060), with 51.6% of those with PMT experiencing difficulties compared to 33.3% without. The effects on attendance (*p* = 0.160), assignment deadlines (*p* = 0.120), and classroom attention (*p* = 0.200) were not statistically significant, with 90.0% of those with PMT and 83.3% of those without reporting difficulties in attendance, 81.1% of those with PMT and 69.4% without in meeting assignment deadlines, and 90.5% of those with PMT and 83.3% without in paying attention in class.

**Table 2:** The relationship between the effect of premenstrual tension syndrome on respondents' academic performance.

Variables	PMT, *n* (%)		*p‐*Value^a^
	No (*n* = 36)	Yes (*n* = 190)	
Effect on attendance			0.160
No	6 (16.7)	19 (10.0)	
Yes	30 (83.3)	171 (90.0)	
Effect on physical activities			0.024
No	11 (30.6)	26 (13.7)	
Yes	25 (69.4)	164 (86.3)	
Effect on assignments due dates			0.120
No	11 (30.6)	36 (18.9)	
Yes	25 (69.4)	154 (81.1)	
Effect on exams			0.060
No	24 (66.7)	92 (48.4)	
Yes	12 (33.3)	98 (51.6)	
Effect on extracurricular activities			0.035
No	11 (30.6)	30 (15.8)	
Yes	25 (69.4)	160 (84.2)	
Effect on paying attention in class			0.200
No	6 (16.7)	18 (9.5)	
Yes	30 (83.3)	172 (90.5)	

Abbreviation: PMT, premenstrual tension.

^a^ Chi square test, *p* value < 0.05 is significant.

## 4 DISCUSSION

Understanding the occurrence and impact of PMS among students in the medical and health science fields is crucial. The demanding nature of their academic curriculum, coupled with the unique challenges posed by PMS, necessitates a comprehensive investigation into this phenomenon. Numerous research studies have already examined the prevalence rates of PMS among university students, including those studying medical and health science disciplines. However, these investigations have yielded a wide range of results, with frequency estimates ranging from approximately 20%–90% among female students.^15^, ^16^, ^17^, ^18^ Consistent with this trend, our study at RAKMHSU has revealed a significant prevalence of PMS among female students, with 78.9% of participants experiencing mild to severe symptoms. It is worth noting that the varying prevalence rates reported in different studies can be attributed to several factors, including the use of different diagnostic criteria for identifying PMS, the assessment methods employed, the cultural and social context of the study population, and geographical location. Furthermore, it is important to recognize that while PMS is widely prevalent, there is a less common but severe subtype known as PMDD, which affects an estimated 3%–8% of women.^4^ Interestingly, this study found a higher prevalence rate of 16.3% for PMDD among participants, surpassing rates reported in other studies.

This elevated prevalence of PMDD among our study participants compared to previous research has significant implications for their academic performance and overall quality of life. Timely identification and treatment of PMDD are crucial as it can have debilitating effects and, if left untreated, may lead to severe mental health issues such as depression and anxiety. Therefore, early recognition and management of PMDD can greatly enhance the quality of life for those affected. Among the symptoms reported in this study, physical symptoms emerged as the primary concern, followed by behavioral symptoms, and then psychological symptoms. The most common psychological symptoms noted were mood swings, depressed mood, and anxiety. These findings are consistent with a 2019 study conducted among college students in Puducherry, which found a prevalence of PMS of 62.7%, lower than the prevalence in the current study.^19^ The previous study also identified physical symptoms such as back, joint, and muscle aches, as well as abdominal discomfort, as the most frequently experienced symptoms.^19^ However, there were differences in the reported psychological symptoms, with affective symptoms being more prevalent. The prevalence of PMS and PMDD in the present study is higher compared to a study conducted at the University of Rio Verde in Brazil, where the rates were found to be 46.9% and 11.1%, respectively.^20^ However, the most commonly reported symptoms in this study were physical, such as breast tenderness, bloating, and weight gain, followed by psychological symptoms, which align with the findings of the current study. A recent study in Nepal also reported a relatively lower prevalence of PMDD among female medical college students, with a rate of 3.8% out of 266 participants.^21^

On the other hand, the prevalence of PMS in the current study is lower than that reported in other studies conducted in India and Jordan, which revealed rates of 86% and 92.3% among female university students, respectively.^22^, ^23^ The most commonly reported psychological symptoms in this study were mood swings, depressed mood, and irritability, while the most common physical symptoms were general body pain, headache, back pain, fatigue, and joint pain. These findings align with the current study's focus on physical symptoms as being most commonly reported among students with PMS.^22^ There has historically been a lack of awareness and recognition of the potential impact of PMS and PMDD on quality of life and daily functioning.^24^ However, in recent years, the medical and mental health communities have started to shift their perception of PMS and PMDD. The inclusion of PMDD in the DSM‐5 by the American Psychiatric Association signifies the recognition of PMDD as a distinct mental health disorder. It should be noted that research on PMS/PMDD and its specific impact on academic performance is still in progress, and further studies are needed to fully understand this relationship.

However, the current study revealed higher percentages of participants experiencing PMT who were absent from class (90%) and avoided social functions (over 80%). In contrast, a study conducted at the School of Nursing, University of Jordan, reported a relatively lower percentage (16%) of participants with low levels of academic involvement.^23^ Similarly, Shamnani et al. found that 12% of individuals with PMS were absent from class and 32% avoided social functions.^18^ These discrepancies in percentages may be influenced by various factors, including the specific characteristics of the study population, the severity of PMS or PMDD symptoms, and the measurement tools used to assess academic involvement and social functions. The findings of the present study indicate that 86.3% of students with PMT do not engage in physical activity, suggesting a potential lack of awareness among female students regarding the benefits of physical activity for managing PMT. Incorporating regular exercise into a healthy lifestyle can have positive impacts on overall well‐being, including during the premenstrual phase. Physical activity has been associated with a range of benefits for individuals experiencing PMS or PMDD symptoms, such as stress reduction, mood improvement, and the release of endorphins.^25^ Moreover, it can assist in weight management and reduce water retention, which is commonly experienced during the premenstrual phase. By increasing awareness and educating female students about the favorable effects of exercise on PMS symptoms, there is a possibility to encourage the integration of regular physical activity not only during the premenstrual period but throughout the entirety of the menstrual cycle. Seeking advice from healthcare professionals, who can offer customized guidance based on individual health conditions and specific PMS symptoms, is recommended. The substantial proportion of respondents (90.0%) in the present study reporting an impact on their academic performance underscores the importance of addressing this issue and implementing appropriate interventions.

### 4.1 Strengths and limitations

This study provides valuable insights into the prevalence and impact of PMT among female students. It highlights the importance of addressing these health concerns to improve overall well‐being and academic performance. The inclusion of a separate pilot study, conducted with a different sample, strengthens the psychometric properties of the questionnaire, confirming its validity and reliability in accurately measuring PMT symptoms among the specific target population. However, there are some limitations to consider when interpreting the study findings. Firstly, the use of convenience sampling from a single institution may limit the external validity of the study, restricting the generalizability of the results to other institutions. Secondly, the nonprobability nature of the sample hampers the generalizability of the findings. It is important to note that the statistical significance tests presented in this manuscript are for illustrative purposes only due to the nonrandomized nature of the sample. Lastly, the use of anonymous self‐administered questionnaires, while protecting participant identity, sometimes led to incomplete reporting, affecting the overall response rate among participants.

## 5 CONCLUSION

In conclusion, this study has revealed a high prevalence of PMS among female students in the UAE. The existence of PMS has been shown to hurt academic engagement, particularly in terms of physical activities and involvement in extracurricular activities. There present study findings have several clinical implications in the management of PMS. However, we recommend further large‐scale studies to explore more accurate impact of PMS among females.

## AUTHOR CONTRIBUTIONS

**Eman Ibrahim Mohamed Omara**: Conceptualization; Data curation; Writing—original draft; Methodology; Formal analysis. **Rasha Aziz Attia Salama**: Conceptualization; Data curation; Writing—original draft; Methodology; Formal analysis. **Talaat Matter Tadross**: Conceptualization; Data curation; Writing—original draft; Methodology; Formal analysis. **Sirwan Khalid Ahmed**: Conceptualization; Supervision; Writing—review and editing; Project administration. **Mona Gamal Mohamed**: Validation; Writing—review and editing. **Syed Masudur Rahman Dewan**: Validation; Writing—review and editing. **Md Rabiul Islam**: Conceptualization; Writing—review and editing; Supervision; Project administration.

## CONFLICT OF INTEREST STATEMENT

The authors declare no conflict of interest.

## TRANSPARENCY STATEMENT

The lead authors Sirwan Khalid Ahmed, Md. Rabiul Islam affirms that this manuscript is an honest, accurate, and transparent account of the study being reported; that no important aspects of the study have been omitted; and that any discrepancies from the study as planned (and, if relevant, registered) have been explained.

## Data Availability

The data that support the findings of this study are available from the corresponding author and secound author upon reasonable request.
